# Supplementary material for: Development and Validation of a Lifestyle Behavior Tool in Overweight and Obese Women through Qualitative and Quantitative Approaches
Source: Nutrients. 2021 Dec 20;13(12):4553. doi: 10.3390/nu13124553 (PMC8707061; doi:10.3390/nu13124553)
Supplement: Supplementary file 1 [file nutrients-13-04553-s001.zip › nutrients-1502983-supplementary.pdf]

Supplementary Figure S1. Participant recruitment flowchart for Pilot Study (Study 2)

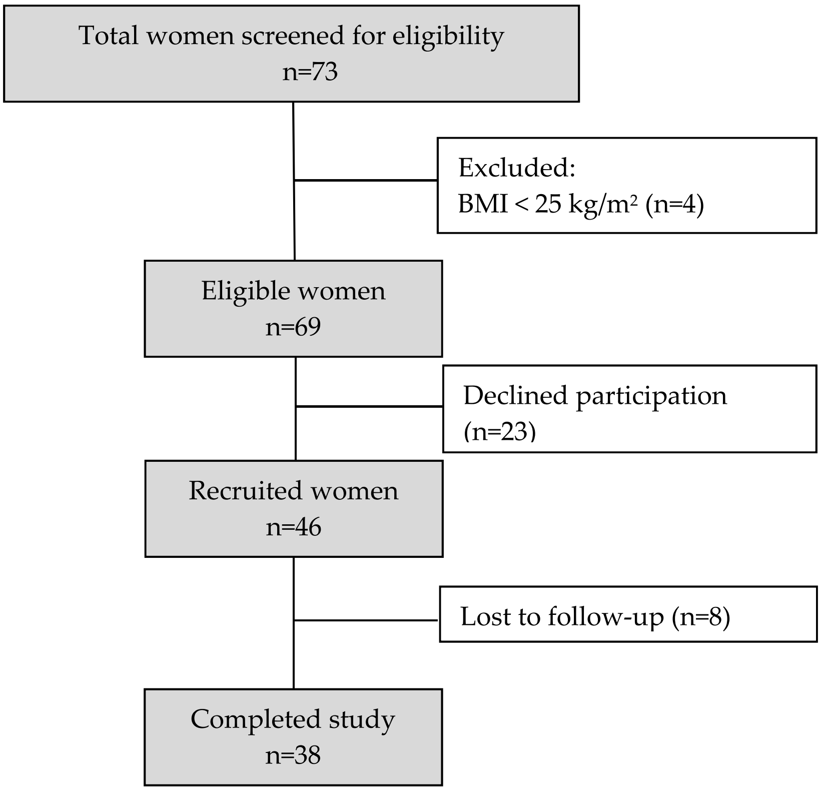

Supplementary Table S1. Sample of the 6P tool

MENTAL MODEL FOR HEALTHY NUTRITION

This instrument is designed to help you understand and monitor YOUR eating patterns, activities and motivational levels in the PAST ONE WEEK.

<table>
<tr><th colspan="3">MAIN MEALS</th></tr>
<tr><td>Over the past week, were your meal timings different on weekdays (working days) compared to weekends (non-working days)?</td><td>Yes | No</td><td rowspan="2">For most days over the past week,
1. I tend to have irregular meal times. (Yes / No)

2. I skipped _____ meals per week.

3. I tend to eat when I felt very hungry. (Yes / No)</td></tr>
<tr><td>Over the past week, what time do you typically consume your meals (e.g. breakfast, lunch, dinner, supper)?

(Weekday / Weekend)</td><td>Weekday: - Time -
Weekend: - Time -

Add meal | Remove meal</td></tr>
<tr><th colspan="3">P1 PORTION</th></tr>
<tr><td>Over the past week, how much grains/high carb foods have you typically eaten for each meal during weekdays (working days) and weekend (non-working days)? Please select one number for each meal to represent food amount taken, at the corresponding time of the meal.</td><td>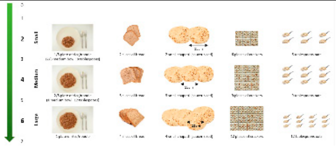
0 refers to no grain / high carbohydrate intake
1 refers to the smallest portion
7 refers to the largest portion</td><td>For most days over the past week,
4. I tend to choose wholegrains / wholemeal options. (Yes / No)

5. I tend to feel very full after a meal. (Yes / No)

6. I tend to finish my meal within 15 min. (Yes / No)</td></tr>
<tr><th colspan="3">P2 PROPORTION</th></tr>
</table>

<table>
<tr>
<td>Over the past week, how much cooked vegetables did you eat for each meal during weekdays (working days) and weekend (non-working days)? Please exclude starchy veggies like potato, sweet potato, yam, pumpkin, tapioca, sweetcorn etc.
Please click the arrows or dots below to select.</td>
<td colspan="2">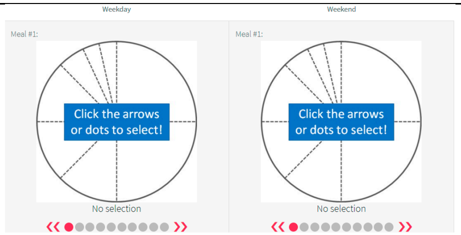
</td>
<td>For most days over the past week,
7. I included fruits as part of my diet. _____ servings per week. (1 serving=1 small apple, orange, mango or pear; 1 wedge papaya, watermelon or pineapple; 10 grapes or longan; 1 medium banana; 1/4 cup dried fruit)

8. I tend to choose vegetables cooked in heavy gravy (e.g. coconut milk, starch, sauces) or deep fried in oil.

(Yes / No)

9. My meals tend to contain deep fried food (e.g. fried meat / seafood, frozen processed meat) and/or dishes cooked in heavy gravy (e.g. coconut milk, starch, sauces). (Yes / No)</td>
</tr>
<tr>
<th colspan="4">P3 PLEASURE</th>
</tr>
<tr>
<td colspan="3">Over the past week, was your snack and beverage consumption different on weekdays (working days) compared to weekends (non-working days)?
Yes | No</td>
<td rowspan="3">For most days over the past week,
10. I tend to snack when not feeling hungry. (Yes / No)

11. I tend to choose snacks that were deep fried and/or sugary. (Yes / No)

12. I tend to snack while watching TV or relaxing. (Yes / No)

13.I tend to choose water as my first choice of drink. (Yes / No)

14. I tend to look for healthier beverage options. (Yes / No)

15. I drank alcohol _____ times per week. (Yes / No)</td>
</tr>
<tr>
<td>Over the past week, when did you typically consume snacks (foods other than the main meals), e.g. chocolate, sweets, chips, nuts, ice-cream, regardless of the amount?</td>
<td>Weekday
- Time -

Add snack | Remove snack</td>
<td>Weekend
- Time -</td>
</tr>
<tr>
<td>Over the past week, when did you typically consume beverages (calorie -containing drinks), e.g. malted drink, soft drink, fruit juice, sugar-sweetened drink, milk, alcohol?</td>
<td>Weekday
- Time -

Add bev. | Remove bev.</td>
<td>Weekend
- Time -</td>
</tr>
</table>

P4 PHASE

Over the past week, what is the distribution of total calorie intake based on the amount of food (including meals, snacks, beverages) you consumed at different timings throughout the day? Instruction: Slide the rectangular bars to represent the distribution (percentage) based on the amount of food you consume at different periods throughout the day.

Weekday

5am-1pm	1pm-7pm	7pm-1am	1am-5am

Weekend

5am-1pm	1pm-7pm	7pm-1am	1am-5am

For most days over the past week,
16. My heaviest meal was usually dinner. (Yes / No)

17. I tend to have my dinner after 9pm. (Yes / No)

18. I tend to eat foods dense in fat / carbohydrate before going to bed (e.g. fast food, instant noodle). (Yes / No)

19. I have felt hungry during my sleep and tend to wake up to snack. (Yes / No)

P5 PHYSICALITY

Over the past week, how many days have you spent on physical activity that takes **moderate effort** and makes you breathe harder than normal (able to talk, but not sing)? (1/2/3/4/5/6/7) **days**

How much time have you spent on average doing such **moderate physical activity** per day? (1/2/3/4/5/ 6) **hours** (0/15/30/45) **min**

Over the past week, how many days have you spent on physical activity that takes **hard effort** and makes you breathe harder than normal (able to talk, but not sing)? (1/2/3/4/5/6/7) **days**

How much time have you spent on average doing such **vigorous physical activity** per day? (1/2/3/4/5/ 6) **hours** (0/15/30/45) **min**

For most days over the past week,
20. I tend to walk for at least 30 minutes a day. (Yes / No)

21. I tend to spend more than 3 hours of screen time a day. (Yes / No)

22. I tend to spend more than 8 hours sitting or lying down a day. (Yes / No)

P6 PSYCHOLOGY

Over the past week, what was your motivational level on changing your eating behavior?<br>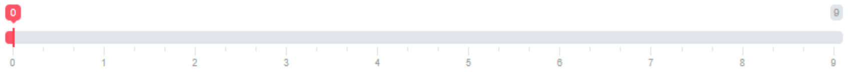 	For most days over the past week,<br>23. I felt changing my eating behavior was challenging. (Yes / No)<br><br>24. I often find it hard to sustain the change. (Yes / No)<br><br>25. I have felt positive when I changed my eating behavior. (Yes / No)

Notes: The appearance of the 6P tool is different in the digital platform due to the built in logic with auto-populated features.

Supplementary Table S2. Sample of feedback report with information on dietary problems derived from the 6P tool, related health implications and recommendations

6P items	Dietary problems	Health implications	Recommendations
P1: Portion	Overeating	Excessive carbohydrates are broken down into glucose, which increases blood sugar level. Excess glucose can be converted and stored as fat in body.	• As part of your 'Healthy Plate', reduce carbohydrate portion proportionately. To help achieve weight loss, reduce intake gradually until your portion is between '1-2'.<br>• Increasing your vegetable intake will help you feel more full.<br>• Use a smaller plate to help better control portion size.
P2: Proportion	High fat intake	Fat has double the number of calories than protein and carbohydrate. Excessive fat in your diet can be easily stored as extra fat in body.	• Limit saturated and trans-fat intake which are commonly found in fried/oily foods, coconut milk, animal fats, processed foods and confectioneries.<br>• Gravy may be a hidden source of fat. Minimise adding gravy to your food.<br>• Healthy fats such as olive, canola and sunflower oil, as well as fatty fish (e.g. salmon, tuna) can be consumed in moderation as part of your balanced diet.
P3: Pleasure	Frequent snacking and unhealthy drink	Frequent snacking and sugary drink intake contribute to extra calorie intake.	• Slowly reduce the frequency of snacking or sugary drink intake to ≤1x/d.<br>• Swap them for healthier choices (e.g. fresh fruits, low fat yoghurt, or handful of unsalted nuts or seeds).
	Irregular intake	Irregular eating pattern disrupts your metabolism and blood sugar level. Meal skipping or delaying tends to cause you to feel excessive hunger, increase your cravings for calorie-dense food and drinks (mainly high in fat & sugar). There's also a tendency to overeat in the next meal.	• There's no strict rule about the optimal number of meals and snacks to consume throughout the day. The key is to be consistent with your daily eating time and frequency.<br>• It's recommended to eat every 4-5h during the day to provide your body with a steady supply of energy, regulate appetite and satiety, which will prevent you from snacking and overeating.

			• If you feel hungry in between meals, opt for fibre-rich and low-calorie food, such as fruits, plain whole meal crackers, whole grain toasts, whole grain cereals or plain/lite microwaved popcorn.
P4: Phase	Predominantly night-time eating	Your metabolism is slower at night. The fall in blood sugar level after eating is lesser at night due to a lower insulin sensitivity than day-time. Eating the majority of total calories at night can predispose to weight gain, obesity and glucose dysregulation.	• Shift a greater proportion of your total calorie intake earlier in the day (before 7pm).<br>• Avoid heavy meals in the 3 hours prior to your bedtime.<br>• Select foods rich in protein and whole grains at night.
P5: Physicality	Physically inactive	If you're not physically active, your metabolism is lower and hence you will burn less calories throughout the day. If you're having knee pain, exercise is usually better for your knee than keeping it still. Not moving your knee may worsen the pain. However, do consult your doctor before embarking on strenuous activity if you've pain in your joints.	• Build up physical activities over time, with a goal of 150 min a week. Aim for at least 30 min of activity per day, in bouts of 10 min or more that can increase your heart rate.<br>• Include activities that make your muscles work harder than usual for at least 2x per week, such as using hand-held weights, resistance bands, carrying groceries, climbing the stairs, yoga and Pilates.
P6: Psychology	Low motivation	Weight loss is a long journey. Obsession on the absolute amount of weight loss can reduce your motivation and that's not ideal.	• Switch the focus by listening to your body and how you feel. Ask yourself 'Am I feeling better when I eat more fruits & vegetables, whole grains, drink more water, exercise?'

Supplementary Figure S2. Samples of the 6P mobile messages in images format. (A) Portion, (B) Proportion, (C) Pleasure, (D) Phase, (E) Physicality, (F) Psychology

A

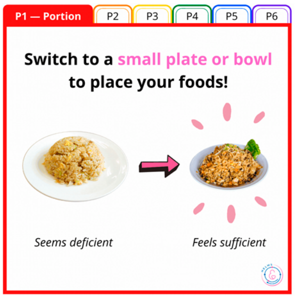

B

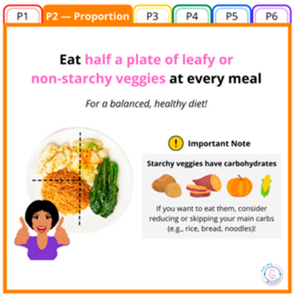

C

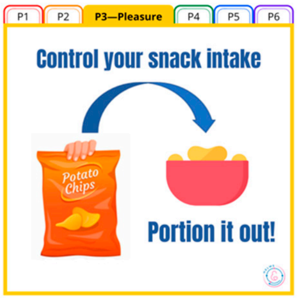

D

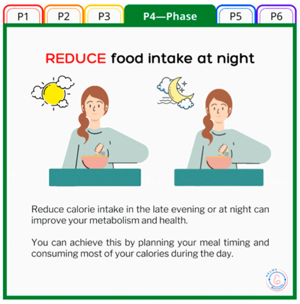

E

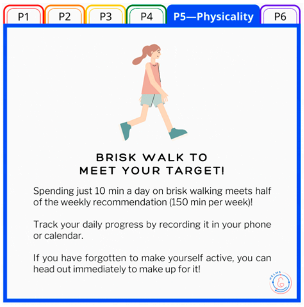

F

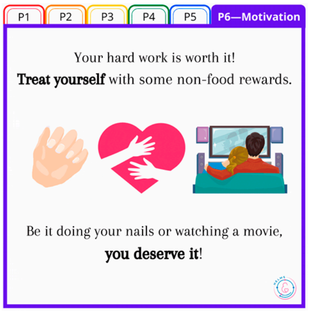

Supplementary Table S3. 6P composite score derivation.

**P1 Portion**<br>Total score for P1 = 0-6	0 if average score is 1-2<br>1 if average score is 0<br>2 if average score is 3-5<br>3 if average score is 6-7<br><br>Average= [(total scores in weekday/number of meals in weekday) + (total scores in weekend/ number of meals in weekend)] /2<br><br>1 if No Q4<br>1 if Yes Q5<br>1 if Yes Q6
**P2 Proportion**<br>Total score for P2 = 0-6	0 if total % >= 50% per day<br>1 if total % >=25 and <50% per day<br>2 if total % >=12.5 to <25% per day<br>3 if total % <12.5% per day<br><br>% per day = (total % in weekday + total % in weekend) /2<br><br>1 if Q7 <14 servings of fruits<br>1 if Yes Q8<br>1 if Yes Q9
**P3 Pleasure**<br>Total score for P3 = 0-12	0 if 0 for total snack and bev per day<br>1 if >0 to <=1 for total snack and bev per day<br>2 if >1 to <3 for total snack and bev per day<br>3 if >= 3 for total snack and bev per day<br><br>Total snack and bev per day = (weekday snack + weekday bev + weekend snack + weekend bev) / 2<br><br>1 if Yes Q1<br>1 if Q2 skip ≥3 meals<br>1 if Yes Q3<br>1 if Yes Q10<br>1 if Yes Q11<br>1 if Yes Q12<br>1 if No Q13<br>1 if No Q14<br>1 if Q15 alcohol > 1
**P4 Phase**<br>Total score for P4 = 0-7	0 if <50% 7pm - 5am<br>1 if ≥50% to <75% 7pm - 5am<br>2 if ≥75% from 7pm - 5am<br>3 if ≥25 % from 1am - 5am<br><br>1 if Yes Q16<br>1 if Yes Q17<br>1 if Yes Q18<br>1 if Yes Q19

**P5 Physicality**<br>Total score for P5 = 0-6	0 if Total ≥ 150 min<br>1 if 120 > Total < 150<br>2 if 75 > Total < 120<br>3 if Total < 75<br><br>Total = (day x moderate activity duration in min) + [(day x vigorous activity in min) x 2]<br><br>1 if No Q20<br>1 if Yes Q21<br>1 if Yes Q22
**P6 Psychology**<br>Total score for P6 = 0-6	0 if P6 score is 7-10<br>1 if P6 score is 5-6<br>2 if P6 score is 3-4<br>3 if P6 score is 1-2<br><br>1 if Yes Q23<br>1 if Yes Q24<br>1 if No Q25

Notes: Total 6P score is 43, with the higher the score, the higher the tendency for unhealthy eating behavior

Supplementary Table S4. 6P tool evaluation survey

6P Tool Evaluation Question	Mean Score ± SD	Disagree[a]/No (%)	Not sure (%)	Agree[b]/Yes (%)
1 (overall presentation)	4.13 ± 0.78	2 (5.3)	3 (7.9)	33 (86.8)
2 (instructions)	4.37 ± 0.67	1 (2.6)	1 (2.6)	36 (94.7)
3 (did not encounter problems)	4.13 ± 0.91	4 (10.5)	1 (2.6)	33 (86.8)
4 (P1 Portion)	4.26 ± 0.72	2 (5.3)	0	36 (94.7)
5 (P2 Proportion)	4.18 ± 0.80	2 (5.3)	0	36 (94.7)
6 (P3 Pleasure)	4.26 ± 0.64	1 (2.6)	1 (2.6)	36 (94.7)
7 (P4 Phase)	4.29 ± 0.61	1 (2.6)	0	37 (97.4)
8 (P5 Physicality)	4.34 ± 0.63	1 (2.6)	0	37 (97.4)
9 (P6 Psychology)	4.34 ± 0.63	1 (2.6)	0	37 (97.4)
10 (P1 Portion self-assessment)	4.24 ± 0.71	2 (5.3)	0	36 (94.7)
11 (P2 Proportion self-assessment)	4.16 ± 0.79	2 (5.3)	0	36 (94.7)
12 (P3 Pleasure self-assessment)	4.29 ± 0.61	1 (2.6)	0	37 (97.4)
13 (P4 Phase self-assessment)	4.29 ± 0.61	1 (2.6)	0	37 (97.4)
14 (P5 Physicality self-assessment)	4.26 ± 0.64	1 (2.6)	1 (2.6)	36 (94.7)
15 (P6 Psychology self-assessment)	4.29 ± 0.61	1 (2.6)	0	37 (97.4)
16 (6P guides healthy eating)	4.13 ± 0.78	1 (2.6)	6 (15.8)	31 (81.6)
17 (6P made me aware of my eating behavior)	4.32 ± 0.62	1 (2.6)	0	37 (97.4)
18 (use 6P to monitor my dietary changes)	4.08 ±0.91	3 (7.9)	2 (5.3)	33 (86.8)
19 (gained new knowledge related to healthy eating)	4.16 ± 0.89	2 (5.3)	3 (7.9)	33 (86.8)
20 (6P recommendations were helpful)	4.08 ± 0.82	1 (2.6)	5 (13.2)	32 (84.2)
21 (how frequent will you fill the 6P tool, weeks)	7.87 ± 8.16			
22 (anything to improve the 6P tool)	-	15 (39.5)	18 (47.4)	5 (13.2)
24 (mobile health messages are helpful)	4.10 ± 0.86	2 (5.3)	3 (7.9)	33 (86.8)
25 (frequency of mobile health messages is just right)	3.97 ± 0.97	5 (13.2)	0	33 (86.8)
26 (preferred frequency of mobile health messages) 1=once a month, 2=once every 2 weeks, 3=once a week, 4=twice a week, 5=thrice a week	3.88 ± 0.99			
27 (anything to improve the mobile health messages)	-	26 (68.4)	10 (26.3)	2 (5.3)
29 (what feature of 6P tool is most useful)				
6P & assessment		14 (36.8)		

6P monitoring chart	16 (42.1)
Nutrition diagnosis & explanation	20 (52.6)
Recommendation report	17 (44.7)
Goal setting	18 (47.4)
Mobile health messages match with selected goals	25 (65.8)

[a]Includes strongly disagree and partially disagree
[b]Includes rather agree and strongly agree
